# Flavonoids from *Scutellaria baicalensis*: Promising Alternatives for Enhancing Swine Production and Health

**DOI:** 10.3390/ijms26083703

**Published:** 2025-04-14

**Authors:** Jing Wu, Yueqin Qiu, Min Tian, Li Wang, Kaiguo Gao, Xuefen Yang, Zongyong Jiang

**Affiliations:** 1 Institute of Animal Science, Guangdong Academy of Agricultural Sciences, Guangzhou 510640, China; wujing@gdaas.cn (J.W.); qiuyueqin@gdaas.cn (Y.Q.); tianmin@gdaas.cn (M.T.); wangli1@gdaas.cn (L.W.); gaokaiguo@gdaas.cn (K.G.); jiangzy@gdaas.cn (Z.J.); 2 State Key Laboratory of Swine and Poultry husbandry Industry, Guangzhou 510640, China; 3 Key Laboratory of Animal Nutrition and Feed Science in South China, Ministry of Agriculture and Rural Affairs, Guangzhou 510640, China; 4 Guangdong Key Laboratory of Animal Husbandry and Nutrition, Guangzhou 510640, China

**Keywords:** *Scutellaria baicalensis* flavonoids, pharmacological effects and mechanisms, network pharmacology analysis, swine management

## Abstract

Concerns over vaccine safety, bacterial resistance, and drug residues have led to increased interest in plant extracts for improving swine nutrition and health. *Scutellaria baicalensis* Georgi, rich in four primary flavonoids—baicalin, baicalein, wogonoside, and wogonin—demonstrates significant pharmacological properties, including anti-inflammatory, antioxidant, antibacterial, and antiviral activities in swine. These flavonoids have been shown to enhance growth performance, improve immunity, modulate gut microbiota, and aid in the prevention and treatment of various diseases. This review highlights the pharmacological effects of these flavonoids in swine, with a focus on network pharmacology to reveal the underlying molecular mechanisms. By constructing drug-target networks and identifying key signaling pathways, the review reveals how these flavonoids interact with biological systems to promote health. Furthermore, it discusses the practical applications of *Scutellaria baicalensis* flavonoids in swine production and outlines potential future research directions. This work provides a theoretical framework for understanding the therapeutic targets of these flavonoids, offering valuable insights for advancing sustainable and healthy pig farming practices.

## 1. Introduction

Pork is a critical source of protein in human diets; however, concerns regarding the safety of its production have increased, particularly due to the misuse of antibiotics and other medications. The overuse of antibiotics in livestock is a significant public health issue, as antibiotic residues can accumulate in the food chain and be ingested by humans. This leads to the development of antibiotic resistance in pathogenic bacteria, posing a substantial threat to global health [1]. As a result, there is an urgent need to identify safe and effective alternatives to antibiotics in animal husbandry.

*Scutellaria baicalensis* Georgi, a perennial herb native to China, is characterized by its robust rhizome, lanceolate leaves, and vibrant purple flowers [2]. In Traditional Chinese Medicine (TCM), the roots of *Scutellaria baicalensis*, known as Huang-Qin, have been used for centuries to treat conditions such as diarrhea, hypertension, and inflammation [3]. These roots are a rich source of bioactive flavonoids, including baicalin, baicalein, wogonoside, and wogonin, which exhibit a range of pharmacological properties, including anti-inflammatory, anticancer, and antioxidant effects [3]. Recent studies have demonstrated that flavonoids extracted from *Scutellaria baicalensis* (SFs) can significantly enhance growth performance and improve disease resistance in pigs [4,5]. However, the underlying mechanisms of SFs remain poorly understood, limiting their broader application in the swine industry.

Based on previous research, and by incorporating network pharmacology analyses, this review aims to summarize the biological activities, mechanisms, and roles of SFs in promoting health in pigs. Additionally, it highlights future research directions and explores the potential applications of SFs in animal production.

## 2. Methods

### 2.1. Predicted Potential Targets of SFs

The 3D structures, PubChem-CID, and SMILES files of flavonoids from SFs were retrieved from the PubChem database (https://PubChem.NCBI.nlm.nih.gov/, accessed on 2 October 2024). To investigate the underlying mechanisms of SFs, a comprehensive analysis was performed using multiple bioinformatics tools. Target genes were identified using PharmMapper (http://www.lilab-ecust.cn/pharmmapper/, accessed on 2 October 2024), Swiss Target Prediction (http://www.swisstargetprediction.ch/), SEA Search Server (http://sea.bkslab.org/, accessed on 2 October 2024), and the STITCH online database (http://stitch.embl.de/). The identified genes were then merged, deduplicated, and integrated into a drug–ingredient–target interaction network using the String database (string-db.org). The network was visualized using Cytoscape 3.10 (https://github.com/cytoscape/cytoscape, accessed on 2 October 2024), a robust tool for the visualization of complex biological data.

### 2.2. Predicted Potential Targets of SFs in Inflammation and Oxidative Stress

Inflammation- and oxidative stress-related target genes were obtained from the GeneCards database (https://www.genecards.org/, accessed on 2 October 2024) and the Comparative Toxicogenomics Database (https://ctdbase.org/, accessed on 2 October 2024). These target genes were merged with those of SFs, deduplicated, and uploaded into Venny (https://www.bioinformatics.com.cn/, accessed on 2 October 2024) to identify the potential targets. The resulting potential targets were then downloaded for further analysis

### 2.3. Construction of Drug–Ingredients–Disease–Targets–KEGG Pathway Network

To perform KEGG pathway enrichment analysis, the potential targets of SFs related to inflammation and oxidative stress were imported into the Database for Annotation, Visualization, and Integrated Discovery (DAVID) (https://david.ncifcrf.gov/) with a threshold of *p* < 0.05. These potential targets were then loaded into a protein–protein interaction (PPI) network diagram using the Search Tool for the Retrieval of Interacting Genes/Proteins (STRING) database (https://www.string-db.org/). *Sus scrofa* was selected as the organism, and a minimum interaction score of 0.9 was set as the threshold. For core target screening and visualization, the TSV-format file was loaded into Cytoscape 3.7.2 (https://github.com/cytoscape/cytoscape/, accessed on 2 October 2024) to construct the drug–ingredients–disease–targets–KEGG pathways network.

## 3. Results and Discussion

### 3.1. Active Ingredients and Target Genes of SFs

**Figure 1:**
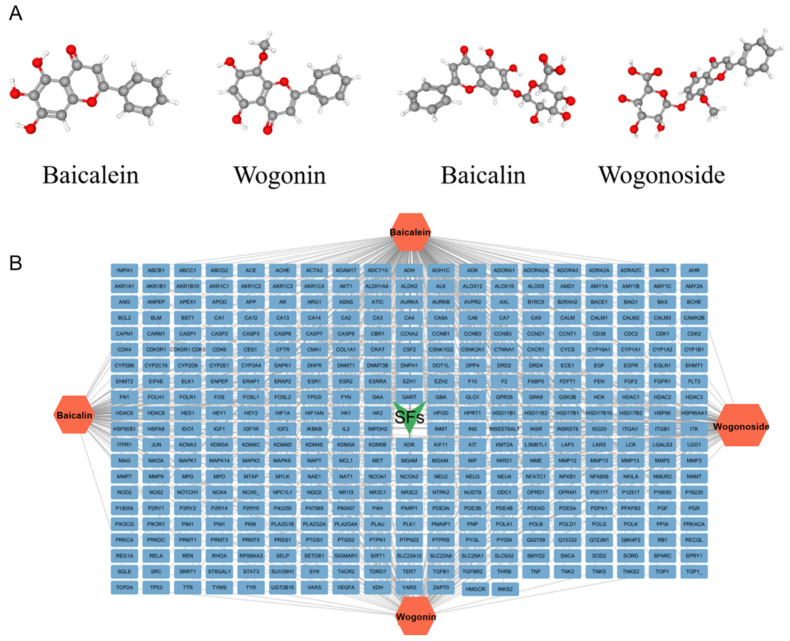
Active ingredients and target genes of SFs. (**A**) SFs’ 3D structure; (**B**) SFs–typical active ingredients–targets network. Green represents the SFs, orange represents the typical active ingredients of SFs, and blue represents the target genes of the active ingredients.

A total of 29 active ingredients from *Scutellaria baicalensis* Georgi (Huangqin), as documented in the TCMSP database, were evaluated according to Lipinski’s Rule of Five [6], as summarized in Table 1. The bioactive components identified in *Scutellaria baicalensis* Georgi include flavonoids, flavonoid glycosides, polysaccharides, trace elements, and volatile oils. Among these, flavonoids and their glycosides are particularly notable for their high content and diverse biological activities [7,8]. Notably, the flavonoids baicalin, wogonoside, baicalein, and wogonin (Figure 1) are widely used in clinical practice due to their pharmacological properties, which include heat-clearing, dampness-drying, fire-resolving, detoxifying, and hemostatic effects [9]. Furthermore, these compounds have garnered significant attention in the livestock industry for their potential applications [3].

Network analysis of the typical active ingredients and targets of SFs (Figure 1) reveals potential molecular targets, providing a comprehensive understanding of the intricate interactions between the bioactive components of SFs and their targets. This analysis offers valuable insights into the therapeutic potential of SFs.

### 3.2. Pharmacological Effects and Mechanisms of SFs

SFs exhibit a broad spectrum of significant medicinal properties and have shown efficacy in various therapeutic areas, including anti-inflammatory [10], antibacterial [11], antiviral [12], antioxidant [13], anticancer [14], and hepatoprotective effects [15]. In this section, we explore the primary pharmacological effects of SFs. To gain insight into the underlying mechanisms, we constructed a network of related pathways using Cytoscape 3.10. This network map clarifies the complex interactions among SFs, as well as their pharmacological effects, molecular targets, and KEGG pathways, providing a solid foundation for further research into the mechanisms of action of SFs.

#### 3.2.1. Anti-Inflammatory Effects

Inflammation is a crucial innate response to tissue injury, primarily aimed at facilitating tissue repair and promoting cell proliferation [16]. SFs, known for their significant anti-inflammatory properties, have been shown to regulate multiple inflammatory cytokines, thereby modulating the inflammatory response [17]. Specifically, baicalin has been demonstrated to reduce inflammation by down-regulating the production of pro-inflammatory cytokines such as interleukin-6 (IL-6), tumor necrosis factor-alpha (TNF-α), interleukin-17 (IL-17), interleukin-1beta (IL-1β), and matrix metalloproteinase-9 (MMP-9) [18]. Additionally, both baicalein and baicalin mitigate inflammation through key signaling pathways, including the NLRP3 inflammasome, toll-like receptor 4 (TLR4), NF-κB, PI3K/Akt/Nrf2, and heme oxygenase 1 (HO-1) [19,20,21]. Wogonin, baicalin, and wogonoside have also been found to significantly reduce lipopolysaccharide (LPS)-induced inflammation [22,23], potentially through mechanisms involving the inhibition of NF-κB and the phosphorylation of p38 mitogen-activated protein kinase (MAPK), while up-regulating heat shock protein 72 (HSP72) [24,25].

**Figure 2:**
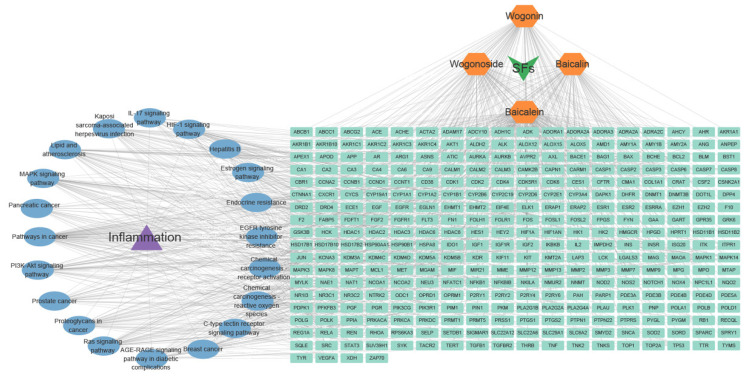
SFs–targets–inflammation–KEGG pathway network. Dark green represents the SFs, yellow represents the typical active ingredients of SFs, purple represents the disease, light green represents the target genes of SFs’ anti-inflammatory effects, and blue represents the KEGG pathway of SFs’ anti-inflammatory effects.

To further explore the anti-inflammatory mechanisms of SFs, we utilized bioinformatics tools such as VENNY (bioinformatics.com.cn) and the STRING database (string-db.org) to analyze the ingredient–target–gene–disease–pathway network. We identified 15,580 inflammation-related target genes from the GeneCards database (https://www.genecards.org/). The analysis revealed 332 potential targets, including six core targets (MAPK3, MAPK1, AKT1, PIK3R1, EGFR, and NF-κB), with a degree greater than 15. These targets were significantly enriched in pathways such as MAPK, HIF-1, C-type lectin receptor, IL-17, and PI3K-Akt signaling (*p* < 3.06 × 10^−12^) (Figure 2). In conclusion, SFs, including baicalin, wogonin, baicalein, and wogonoside, exert anti-inflammatory effects through multiple pathways, suggesting that they have potential as natural therapeutics for inflammatory diseases.

#### 3.2.2. Antioxidant Effects

Oxidative stress is a major factor contributing to cellular damage, and antioxidants play a critical role in mitigating this harmful process. The antioxidant activity of SFs has been extensively documented, providing protection against oxidative stress-induced damage. SFs exhibit their antioxidant effects through various mechanisms, including inhibition of lipid peroxidation, activation of antioxidant enzymes, free radical scavenging, metal ion complex formation, and elimination of reactive oxygen species (ROS) [26,27]. Notably, the flavonoid compounds baicalin and baicalein demonstrate pronounced antioxidant activity, attributed to their phenolic hydroxyl groups [28,29]. Research has shown that these compounds protect neuronal cells from peroxynitrite anion-induced apoptosis by modulating the expression of 12/15-lipoxygenase (12/15-LOX), reducing mitochondrial ROS production, regulating the Bcl-2–Bax ratio, and inhibiting cytochrome c release [30,31,32,33]. Among these compounds, baicalein is reported to possess superior antioxidant potency compared to baicalin [34]. Furthermore, baicalein’s protective effect against oxidative stress-induced apoptosis is enhanced through activation of the PI3K/Akt pathway [35,36,37].

The antioxidant effects of SFs extend beyond the central nervous system to include skin protection, where they mitigate ultraviolet-induced oxidative damage by scavenging free radicals and resisting lipid peroxidation. This highlights their potential value in dermatological applications [38,39]. The correlation between the antioxidant activity of SFs and their total phenolic content underscores their potential as natural antioxidants in the food industry [26,40]. Additionally, SFs show promise for treating diseases induced by oxidative stress, such as diabetes and steatohepatitis [41]. Baicalin and baicalein are particularly effective in reducing oxidative stress, promoting the expression of antioxidant-related genes, and protecting cells by modulating the Nrf2 pathway [42,43].

**Figure 3:**
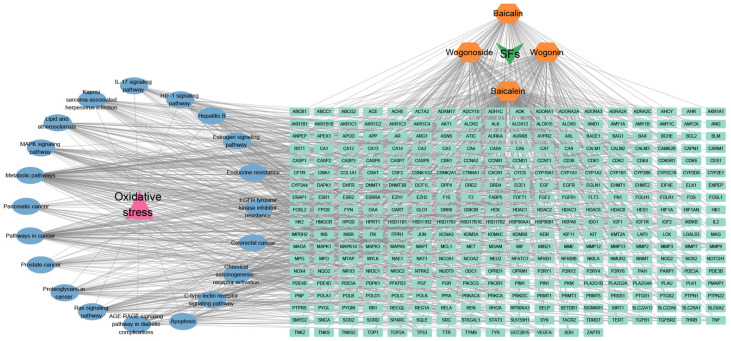
SFs–targets–oxidative stress–KEGG pathway network. Yellow represents the typical active ingredients of SFs, pink represents the disease, light green represents the target genes of SFs’ antioxidant effects, and blue represents the KEGG pathway of SFs’ antioxidant effects.

Although extensive research has been conducted on the antioxidant activity of SFs, a comprehensive understanding of the molecular mechanisms requires further investigation. To address this, we performed a network pharmacology analysis to systematically elucidate the targets and pathways associated with the antioxidant effects of SFs. Our analysis identified 337 potential targets from a pool of 13,633 oxidative stress-related genes, including MAPK1, MAPK3, AKT1, PIK3R1, RELA, EGFR, and NF-κB (degree ≥ 15), which were significantly enriched in various pathways, such as lipid metabolism, atherosclerosis, MAPK, HIF-1, C-type lectin receptor, EGFR tyrosine kinase inhibitor resistance, and apoptosis (*p* < 3.40 × 10^−12^) (Figure 3). These findings highlight the multifaceted antioxidant mechanisms of SFs and their potential therapeutic applications.

#### 3.2.3. Antibacterial Effects

SFs are widely recognized for their antibacterial activity. Baicalein, wogonin, and baicalin have shown significant inhibitory effects on a range of common pathogenic bacteria, including *Aeromonas hydrophila*, *Edwardsiella tarda*, *Vibrio alginolyticus*, *Vibrio harveyi*, *Cariogenic bacteria*, *Bacillus subtilis*, *Enterococcus faecalis*, *Klebsiella pneumoniae*, *Salmonella enterica*, and *Escherichia coli* [44,45,46,47]. These flavonoids also exhibit antimycotic activity against fungi such as *Rhodotorula rubra, Candida albicans, Aspergillus fumigatus,* and *Geotrichum candidum* [48]. Direct bactericidal effects of SFs have been documented against *Helicobacter pylori* and *Staphylococcus aureus* [49,50]. Furthermore, SFs demonstrate synergistic effects with certain antibiotics against resistant strains, such as enhancing activity against methicillin-resistant *Staphylococcus aureus* (MRSA) when combined with ciprofloxacin, ceftriaxone, gentamicin, and penicillin [51,52].

The antibacterial mechanisms of SFs are multifaceted. For example, baicalein inhibits *Staphylococcus aureus* by disrupting bacterial biofilm formation and reducing the secretion of staphylococcal enterotoxin A and α-hemolysin [53]. It also diminishes virulence by binding directly to the major virulence factor of *S. aureus,* the von Willebrand factor-binding protein (vWbp), at Asp-75 and Lys-80 residues [54]. Both baicalin and baicalein disrupt nucleic acid formation and alter bacterial energy metabolism, inhibiting bacterial proliferation [55]. Baicalin’s interaction with cell wall synthesis proteins, such as 1UAG and 2X5O, demonstrates its ability to inhibit cell wall synthesis, disrupt cell wall integrity, reduce bacterial enzyme activity, and inhibit nucleotide synthesis [56].

In addition to the direct effects on bacterial proliferation and virulence factors, SFs also demonstrate antibacterial efficacy by targeting various molecular pathways to regulate the host immune response (Table 2). For example, SFs inhibit the activity of extra-intestinal pathogenic *Escherichia coli* (ExPEC), enterotoxigenic *E. coli* (ETEC), *E. coli* K88, *Haemophilus parasuis,* and *Lactobacillus* by inhibiting NF-κB/MAPK signaling pathways and reducing activation of the NLRP3 inflammasome [57,58,59,60,61,62]. Furthermore, SFs influence antibacterial activities by inhibiting the PANX-1/P2Y6 signaling pathway, disrupting the LuxS/AI-2 quorum-sensing system, and attenuating biofilm formation, which modulates virulence factor expression. SFs also protect vascular tight junctions, reduce ROS production, and modulate apoptosis [63,64,65,66]. Moreover, SFs alter the expression profiles of microRNAs, long non-coding RNAs, and mRNAs, suggesting their potential role in gene regulation [67,68,69].

Collectively, these mechanisms underscore the potential of SFs as broad-spectrum antibacterial agents, capable of modulating both bacterial and host immune responses, to effectively combat infections. Further research is needed to fully elucidate these mechanisms and explore the therapeutic potential of SFs in promoting healthy pig husbandry.

#### 3.2.4. Antiviral Effects

SFs have demonstrated therapeutic potential against a wide range of viral pathogens, including the influenza A virus [72], severe acute respiratory syndrome coronavirus 2 (SARS-CoV-2) [73], Zika virus [74], dengue virus [75], and various arboviruses [76]. Furthermore, the immunomodulatory effects of SFs on porcine reproductive and respiratory syndrome virus (PRRSV) and pseudorabies in piglets have been documented [77,78]. However, research on the antiviral effects of SFs in swine husbandry remains in its early stages.

**Figure 4:**
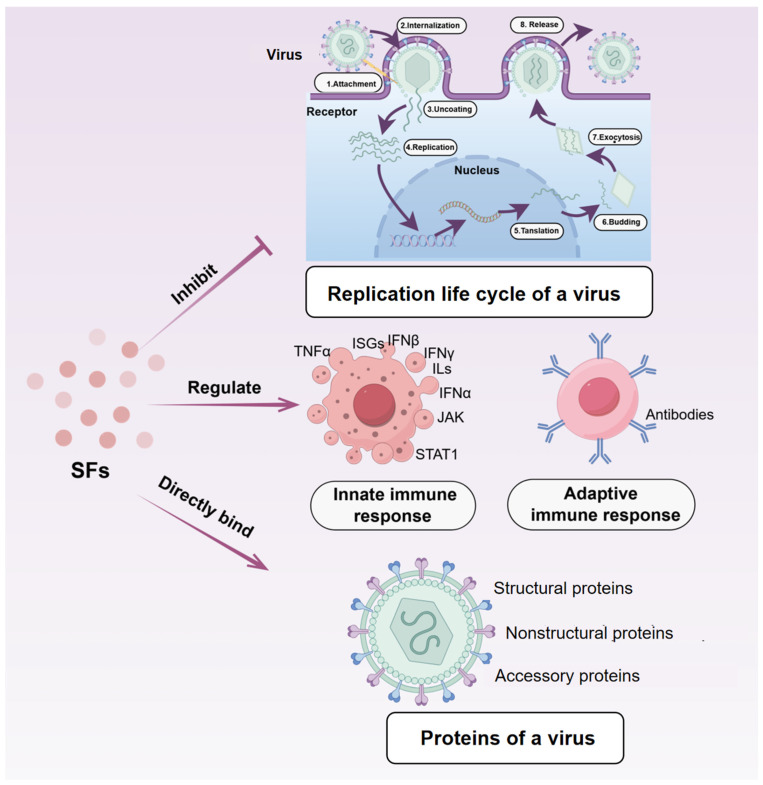
The underlying antiviral mechanisms of SFs include direct interference with the viral life cycle, improvement in host immunity, and direct binding to viral proteins.

The antiviral mechanisms of SFs involve direct interference with the viral life cycle, enhancement of host immunity, and direct binding to viral proteins [79] (Figure 4). These compounds alleviate pathological manifestations induced by viral infections by modulating the host immune response, which includes regulation of interferon and pro-inflammatory cytokine production, such as TNF-α, IFN-α, IFN-γ, and various interleukins [80]. Baicalin, recognized as a non-nucleoside reverse transcriptase inhibitor with anti-HIV-1 properties, obstructs HIV-1 entry into host cells by disrupting the interaction between the virus and the cell surface receptors [81,82]. Baicalein, a derivative of baicalin [83], induces a conformational change by binding to the hydrophobic region within the catalytic core of integrase, acting as an inhibitor of HIV-1 integrase [84]. Notably, baicalein’s inhibitory effect on HIV-1 reverse transcriptase is four times greater than that of baicalin [83].

In summary, this section emphasizes the substantial research potential of SFs in antiviral therapy. However, further research is needed to fully elucidate the specific antiviral mechanisms and expand their applications, especially concerning porcine viral diseases.

### 3.3. Application of SFs in Swine Health Management

In addition to their pharmaceutical applications, SFs play a crucial role in enhancing pig health as feed additives, as summarized in Table 3.

#### 3.3.1. Improvement in Growth Performance

The inclusion of SFs in feed additives has shown promising results in enhancing the growth performance of swine (Table 3). For example, a study involving weaned piglets aged 28 to 56 days reported that the administration of fermented *S. baicalensis*, a derivative of SFs, at a concentration of 1.5 mg/kg of diet, improved appetite, average daily feed intake, and feed conversion efficiency. It also reduced the feed-to-weight ratio and diarrhea rate [85]. Additionally, the inclusion of mixed fermented plants containing SFs in the diets of growing pigs has been shown to improve growth performance, significantly enhance nutrient digestibility, and reduce the emission of noxious gases from excreta [86,87]. The addition of SFs to the diets of pregnant and lactating sows has also been observed to reduce weight loss post-delivery and improve litter performance [88]. In reproductive health, baicalin has been found to enhance the development of both parthenogenetically stimulated and in vitro fertilized pig embryos by modulating mitochondrial activity, stimulating the sonic hedgehog signaling pathway, preventing reactive oxygen species generation, and inhibiting apoptosis [97]. These findings collectively demonstrate the potential of SFs to optimize nutritional management and health in pigs, leading to improved overall growth performance. The application of SFs in feed additives represents a significant advancement in swine nutrition and health that warrants further research and development.

#### 3.3.2. Enhancement of Immunity

SFs have shown significant immunomodulatory effects in swine health management, enhancing the immune systems of weaned piglets, thereby reducing infection rates and improving overall health (Table 3). For example, baicalein has demonstrated considerable benefits in preventing infections, boosting immunity, and modifying the intestinal microbial composition in 28-day-old weaned piglets [89]. In another study, the administration of baicalin at a concentration of 1 g/kg of feed for the first week, followed by 500 mg/kg for the next two weeks, was found to alleviate intestinal injury in 21-day-old weaned piglets, suggesting a protective role against intestinal inflammation [90]. Additionally, feeds containing SFs have been shown to offer preventive benefits against *Salmonella* infections in piglets due to their anti-inflammatory properties [89]. These studies collectively highlight SFs’ potential as immunomodulators in the dietary management of pigs, especially during the critical early weaning stage when their immune systems are not yet fully developed. The strategic use of SFs at this stage can provide essential support for the health and development of these young animals, emphasizing the importance of further research and optimization of SFs in swine nutrition and health management.

#### 3.3.3. Regulation of the Intestinal Microbiome

The modulation of the gut microbiome is a key aspect of swine health management, and SFs have emerged as effective feed additives in this process (Table 3). Research has shown that SFs significantly influence the composition of the intestinal microbiota. For instance, administering a mixed fermented plant containing SFs to weaned piglets at 25 days of age has been demonstrated to alter gut microbial composition, which may contribute to improved ether extract digestibility, gut health, and overall health status [5]. Furthermore, SFs have shown protective effects against intestinal injury caused by *E. coli* K88 in weaned piglets, exerting their effects through the NF-κB/P38 signaling pathways [58]. Additionally, administering baicalin–aluminum complexes at a dosage of 1.36 g/day for three days has been found to modulate the gut microbiomes of 10-day-old piglets, suggesting a role in maintaining gut homeostasis [91].

These findings underscore the potential of SFs to foster a healthy gut environment, which is essential for preventing gastrointestinal disorders and enhancing overall health in swine. The capacity of SFs to modulate the gut microbiome highlights their significance as a dietary supplement in swine nutrition, promoting the development of robust and diverse microbial communities that are essential for animal health and well-being.

#### 3.3.4. Disease Prevention and Treatment

SFs have been extensively studied for their therapeutic potential in treating various diseases in pigs, owing to their anti-inflammatory, antioxidant, antibacterial, and antiviral properties. As detailed in Table 2, SFs have demonstrated significant efficacy in the swine industry, particularly against bacterial infections. Baicalin, a key component of SFs, has proven effective in preventing and treating infection with *Haemophilus parasuis*, a common cause of Glässer’s disease in pigs, which is characterized by vascular inflammation and damage [68,69,70]. Fermented plants and traditional Chinese medicine formulations containing SFs also play a crucial role in reducing the incidence of diarrhea in pigs, due to their regulatory effects on intestinal health and potent antimicrobial activity [60,92,93,98]. In the treatment of edema disease in weaned piglets aged 28 to 35 days, baicalein injections at doses of 0.2 and 0.4 mL/kg have achieved therapeutic efficacy rates as high as 90% [94], indicating that they are effective in treating edema in weaned piglets. In vitro experiments have shown that baicalin, at concentrations of 5–20 μg/mL, significantly inhibits the porcine reproductive and respiratory syndrome virus (PRRSV) in a dose-dependent manner, through the regulation of antiviral cytokine expression and direct interaction with virions, thereby affecting various stages of the virus life cycle [77]. Additionally, polysaccharides from *Scutellaria baicalensis* (SGP) have exhibited therapeutic effects against pseudorabies virus (PRV) infection in piglets by reducing clinical symptoms and pathological damage while enhancing T lymphocyte conversion [78].

In summary, the broad-spectrum pharmacological effects of SFs underscore their potential as valuable therapeutic feed additives for pig diseases. Their ability to regulate immune responses, improve intestinal health, and directly target pathogens positions SFs as promising candidates for advancement in veterinary medicine, potentially serving as alternatives to antibiotics in feed additives.

## 4. Conclusions and Future Directions

SFs demonstrate a broad spectrum of pharmacological properties that effectively enhance porcine growth performance and overall health status, consequently improving production efficiency and economic viability in swine operations. Experimental evidence reveals that the administration of SFs significantly improves intestinal barrier integrity and modulates immune responses, thereby reducing disease susceptibility and associated veterinary expenditures. Their demonstrated efficacy as natural antimicrobial and anti-inflammatory compounds enables partial substitution of conventional antibiotics in feed formulations, addressing critical concerns regarding antimicrobial resistance development while meeting increasing market demand for antibiotic-free animal products. From an environmental perspective, supplementation with SFs contributes to ecological sustainability through reduced antibiotic residues in effluents, thereby mitigating soil and water contamination risks. These multifaceted advantages position SFs as promising phytogenic additives for sustainable swine production systems, demonstrating considerable potential for practical applications in modern intensive farming systems.

Despite their demonstrated anti-inflammatory, antibacterial, antiviral, and antioxidant properties, the clinical application of SFs is currently limited due to their low bioavailability. To overcome this challenge, advanced technologies such as high-throughput screening should be employed to rapidly identify the bioactive constituents of SFs with therapeutic potential. Additionally, bioinformatics and proteomics can provide deeper insights into the efficacy and safety of SFs. Optimizing the extraction process, innovating SF formulations in conjunction with other TCM components, and exploring modern production methods such as ultra-micro grinding technology and TCM fermentation, are crucial for developing more effective products. Furthermore, enhancing the solubility, stability, and bioavailability of SFs’ secondary metabolites, as well as improving their palatability as feed additives, is essential for their broader application in animal husbandry.

SFs have emerged as important regulators of lipid metabolism and fat synthesis in animals. While data regarding their effects on fat metabolism in pigs are limited, studies indicate that baicalin and baicalein possess anti-hyperlipidemic properties by modulating pathways such as PPAR signaling, glycerolipid metabolism, and cholesterol biosynthesis [99,100]. Additionally, SFs have been shown to reduce triglyceride levels, prevent hepatic fat accumulation, and improve insulin resistance through the AMPK-mediated SREBP pathway [101,102]. SFs have also demonstrated potential in mitigating metabolic dysregulation caused by high-fat and high-sugar diets [103]. These findings suggest that SFs may have significant effects on lipid metabolism and fat synthesis in pigs, warranting further investigation.

As a natural antiviral agent, SFs hold promise for mitigating the emergence of drug resistance and modulating the host immune system. Future research should focus on exploring the application of SFs in the prevention and control of swine diseases, integrating the concepts of “prevention before disease onset” and “medicine–food homology.” Incorporating SFs into modern husbandry practices, such as feed additives, could reduce the stress associated with vaccination and drug treatment while enhancing pig immunity and disease resistance. Moreover, understanding the mechanisms underlying the antibacterial and antiviral actions of SFs is critical for evaluating their potential as antibiotic-free feed additives and disinfectants. This would integrate the traditional properties of SFs with modern scientific advancements, offering innovative solutions for sustainable and healthy pig husbandry.

In conclusion, this review has emphasized the significant role of SFs in promoting healthy pig husbandry. We have highlighted their active ingredients, target genes, pharmacological effects, signaling pathways, and practical applications. By elucidating the underlying mechanisms of SFs, we aim to provide guidelines for their integration into the development of healthier and more sustainable pig husbandry practices.

## Figures and Tables

**Table 1:** Active ingredients of *Scutellaria baicalensis* Georgi.

Mol ID	Molecule Name	Structure	MW	Alogp	Hdon	Hacc	OB (%)	DL
MOL001689	Acacetin	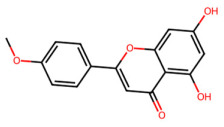	284.28	2.59	2	5	34.97	0.24
MOL000173	Wogonin	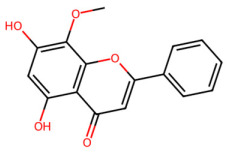	284.28	2.59	2	5	30.68	0.23
MOL000228	(2R)-7-hydroxy-5-methoxy-2-phenylchroman-4-one	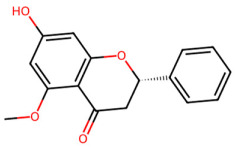	270.3	2.82	1	4	55.23	0.2
MOL002714	Baicalein	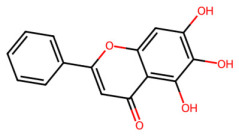	270.25	2.33	3	5	33.52	0.21
MOL002908	5,8,2′-Trihydroxy-7-methoxyflavone	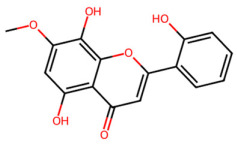	300.28	2.32	3	6	37.01	0.27
MOL002909	5,7,2,5-tetrahydroxy-8,6-dimethoxyflavone	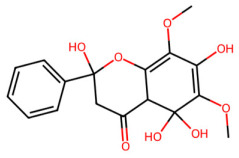	376.34	2.02	4	9	33.82	0.45
MOL002910	Carthamidin	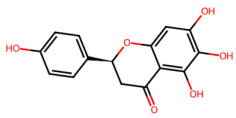	288.27	2.03	4	6	41.15	0.24
MOL002911	2,6,2′,4′-tetrahydroxy-6′-methoxychaleone	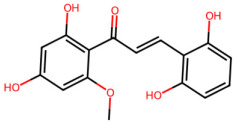	302.3	2.62	4	6	69.04	0.22
MOL002913	Dihydrobaicalin_qt	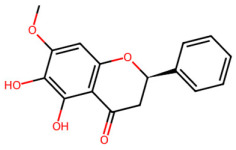	272.27	2.3	3	5	40.04	0.21
MOL002914	Eriodyctiol	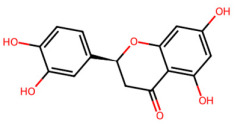	288.27	2.03	4	6	41.35	0.24
MOL002915	Salvigenin	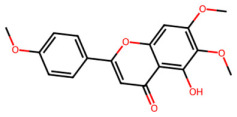	328.34	2.82	1	6	49.07	0.33
MOL002917	5,2′,6′-Trihydroxy-7,8-dimethoxyflavone	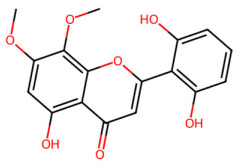	330.31	2.3	3	7	45.05	0.33
MOL002925	5,7,2′,6′-Tetrahydroxyflavone	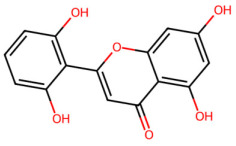	286.25	2.07	4	6	37.01	0.24
MOL002926	Dihydrooroxylin A	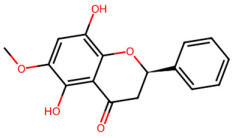	286.3	2.55	2	5	38.72	0.23
MOL002927	Skullcapflavone II	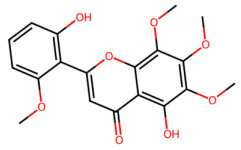	374.37	2.54	2	8	69.51	0.44
MOL002928	Oroxylin a	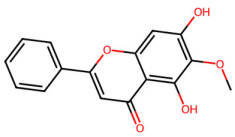	284.28	2.59	2	5	41.37	0.23
MOL002932	Panicolin	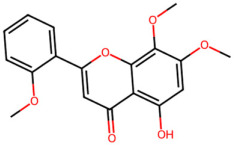	314.31	2.57	2	6	76.26	0.29
MOL002933	5,7,4′-Trihydroxy-8-methoxyflavone	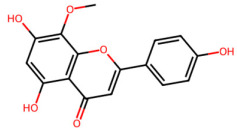	300.28	2.32	3	6	36.56	0.27
MOL002934	Neobaicalein	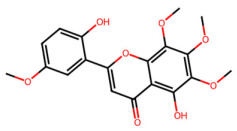	374.37	2.54	2	8	104.34	0.44
MOL002937	Dihydrooroxylin	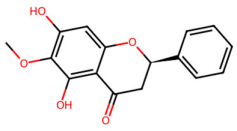	286.3	2.55	2	5	66.06	0.23
MOL000525	Norwogonin	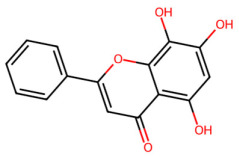	270.25	2.33	3	5	39.4	0.21
MOL000552	5,2′-Dihydroxy-6,7,8-trimethoxyflavone	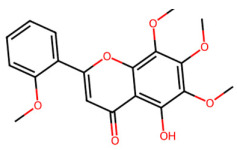	344.34	2.55	2	7	31.71	0.35
MOL000073	Epicatechin	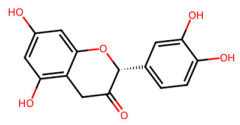	290.29	1.92	5	6	48.96	0.24
MOL001458	Coptisine	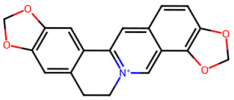	320.34	3.25	0	4	30.67	0.86
MOL002897	Epiberberine	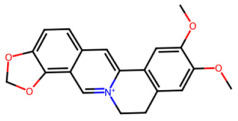	336.39	3.45	0	4	43.09	0.78
MOL008206	Moslosooflavone	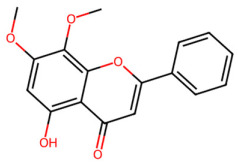	298.31	2.84	1	5	44.09	0.25
MOL012245	5,7,4′-trihydroxy-6-methoxyflavanone	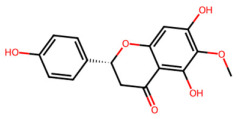	302.3	2.28	3	6	36.63	0.27
MOL012246	5,7,4′-trihydroxy-8-methoxyflavanone	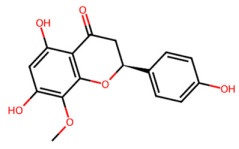	302.3	2.28	3	6	74.24	0.26
MOL012266	Rivularin	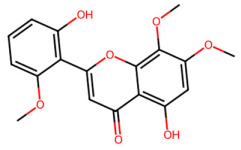	344.34	2.55	2	7	37.94	0.37

Note: Mol ID, molecule ID; MW, molecular weight; Alogp, lipophilicity; Hdon, hydrogen bond donor; Hacc, hydrogen bond acceptor; OB, oral bioavailability; DL, drug-likeness.

**Table 2:** Antibacterial activities of SFs in the swine husbandry industry.

Compound	Antibacterial Activity	Effective Concentration/MIC	Related Mechanism	Reference
Baicalin	ETEC	1 µg/mL	Inhibits bacterial adhesion and activates NF-κB signaling pathway	[19]
Baicalin	*H. parasuis*	12.5–100 μg/mL	Suppresses NLRP3 inflammasome and NF-κB signaling	[57]
SBE (≥ 12% baicalin)	*E. coli* K88	1000 mg/kg BW	Inhibits NF-κB and P38 signaling pathways	[58]
GQD	*Lactobacillus*	1.22 g/kg	Inhibits TLR2/MyD88/ NF-κB pathway	[60]
Baicalin	ExPEC	1600 µg/mL	Inhibits LuxS/AI-2 quorum-sensing system	[61]
Baicalin	ExPEC	1600 µg/mL	Inhibits the expression of NF-κB/MAPK signaling pathways and reduces NLRP3 inflammasome activation	[62]
Baicalin	*G. parasuis*	25–100 µM	Inhibits PANX-1/P2Y6 signaling pathway	[63]
Baicalin	*H. parasuis*	12.5–100 μg/mL	Modulates apoptosis via RAGE, MAPK, and AP-1	[64]
Baicalin	*G. parasuis*	25–100 mg/kg BW	Protects vascular tight junctions	[65]
Baicalin	*S.aureus*	760 μg/mL	Attenuates biofilm formation and downregulates expression of virulence-related factors	[66]
Baicalin	*G. parasuis*	50 μg/mL	Changes lncRNA expression	[67]
Baicalin	*H. parasuis*	50 μg/mL	Changes microRNA expression profiles	[68]
Baicalin	*H. parasuis*	50 μg/mL	Changes expression profiles of long non-coding RNAs and mRNAs	[69]
Baicalin	*H. parasuis*	80 mg/kg·BW	Reverses apoptosis through regulating PKC-MAPK signaling pathway	[70]
Baicalin	*G. parasuis*	25–100 mg/kg BW	Inhibits activation of HMGB1, cell apoptosis, and MAPK signaling pathway	[71]

BW, body weight; ExPEC, extra-intestinal pathogenic Escherichia coli; ETEC, enterotoxigenic Escherichia coli; S. aureus, Staphylococcus aureus; SBE, Scutellaria baicalensis extracts; E. coli K88, Escherichia coli K88; G. parasuis, Glaesserella parasuis; H. parasuis, Haemophilus parasuis; GQD, Gegen Qinlian decoction.

**Table 3:** The application of SFs in healthy swine husbandry.

Compound	Effective Concentration	Type of Animal	Age	Main Effects	References
Mixed herbs containing *S. baicalensis*	1000 mg/kg	Sows and suckling piglets	80–107 d of gestation	Improves antioxidant capacity, liver function, colostrum quality, and immunity in sows; improves growth performance and immunity in piglets	[4]
Mixed fermented plant containing *S. baicalensis*	-	Weaned piglets	25 d	Modifies gut microbial composition	[5]
*Scutellaria baicalensis* extracts	1000 mg/kg diet	Weaned piglets	24 ± 2 d	Attenuates diarrhea	[58]
Baicalin–aluminum complexes	1.36 g/d, 3 d	Piglets	10 d	Modulates gut microbiome composition of piglets with diarrhea	[64]
Baicalin	5–20 μg/mL	MARC145 cells and PAMs	-	Inhibits PRRSV infection in vitro	[77]
Fermented *S. baicalensi*	1.5 mg/kg diet	Weaned piglets	28–56 d	Enhances appetite, increases average daily intake, reduces feed-to-weight ratio and diarrhea rate, and improves feed reward	[85]
Mixed fermented plant containing *S. baicalensis*	-	Growing pigs	25.50 ± 2.50 kg BW	Improves growth performance and nutrient digestibility; reduces noxious gas emissions of excret	[86]
Mixed herbs containing *S. baicalensis*	0.025% and 0.05%	Finishing pigs	44.2 ± 2.23 kg BW	Improves growth performance and nutrient digestibility; decreases serum cortisol levels; benefits meat quality	[87]
Mixed herbs containing *S. baicalensis*	-	Pregnant and lactating sows	-	Decreases weight loss and improves litter performance	[88]
Mixed herbs containing Baicalin	89.74 mg/g	Weaned piglets	28 d	Prevents infection, enhances immunity, and adjusts intestinal microbial composition	[89]
Baicalin	1 g/kg for week and 500 mg/kg feed for 2 weeks	Weaned piglets	21 d	Alleviates intestinal injury	[90]
Baicalin	100 μg/mL	Pig intestinal epithelial cell line J2	-	Attenuates intestinal inflammatory injury	[91]
Mixed herbs containing *S. baicalensis*	10 g/d	Lactating sows under heat stress and piglets	-	Improves feed intake, digestibility of dry matter, piglets’ weaning weight, and ADG; decreases backfat loss, serum cortisol levels, and diarrhea	[92]
*S. baicalensis* extract	-	Weaned piglets	-	Improves growth performance and nutrient digestibility; decreases fecal noxious gas emissions and alleviates diarrhea	[93]
Baicalin	10 mg/kg BW, 1 time/d, 5 d; intramuscular injection	Weaned piglets	28–35 d	Prevents swine edema disease	[94]
Baicalin	500 mg/kg	Weaned piglets	-	Restores intestinal health	[95]
Mixed herbs containing *S. baicalensis*	1000 mg/kg	Sows and suckling piglets	-	Improves growth performance, maternal metabolism, and transmission of antibodies	[96]

BW, body weight; PAMs, porcine alveolar macrophages.

## Abbreviations

SFs	*Scutellaria baicalensis*’s Flavonoids
ADG	Average Daily Gain
ADFI	Average Daily Feed Intake
KEGG	Kyoto Encyclopedia of Genes and Genomes
MAPK	Mitogen-Activated Protein Kinase
AKT	Protein Kinase B
PI3K	Phosphoinositide 3-Kinase
EGFR	Epidermal Growth Factor Receptor
NF-kB	Nuclear Factor kappa-light-chain-enhancer of Activated B cells
NLRP3	NOD-like Receptor Family Pyrin Domain Containing 3
ROS	Reactive Oxygen Species
HIV	Human Immunodeficiency Virus
TCM	Traditional Chinese Medicine
